# Supplementary figures and images for: Systematic and scalable genome-wide essentiality mapping to identify nonessential genes in phages
Source: PLoS Biol. 2023 Dec 4;21(12):e3002416. doi: 10.1371/journal.pbio.3002416 (PMC10695390; doi:10.1371/journal.pbio.3002416)

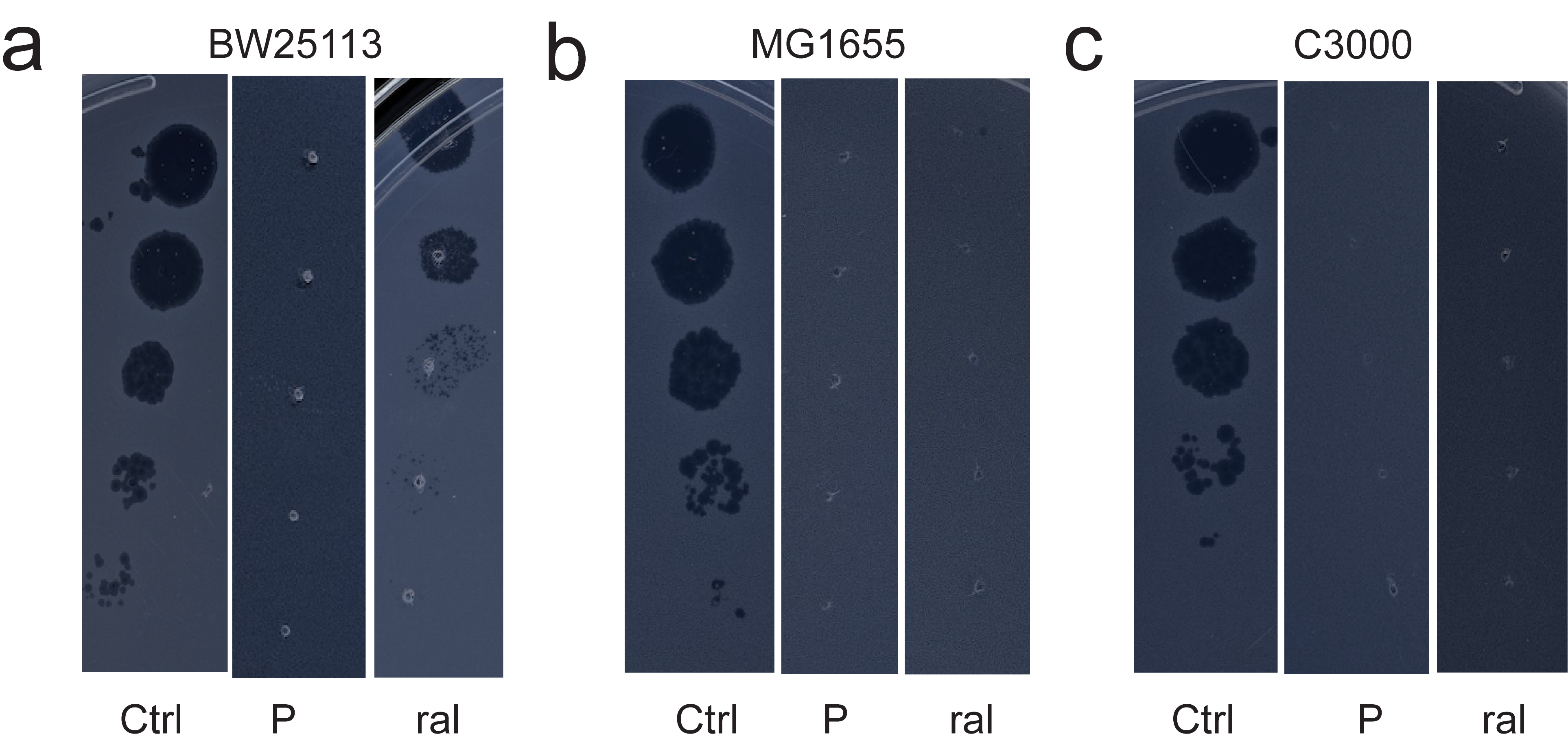

Supplement: S2 Fig — (a) EOP experiments with crRNA targeting ral in E. coli BW25113 (methods). (b) EOP experiments with crRNA targeting ral in E. coli MG1655 that has an active type 1 restriction modification system. (c) EOP experiments with crRNA targeting ral in E. coli C3000 that has an active type 1 restriction modification system. For comparison, phage plaques appearing on E. coli lawn expressing a crRNA targeting essential gene P and nontargeting crRNA (targets P1 phage mcp) as a control are shown for lambda phage (Ctrl). (TIF) [file pbio.3002416.s002.tif]
