# Supplementary material for: Systematic and scalable genome-wide essentiality mapping to identify nonessential genes in phages
Source: PLoS Biol. 2023 Dec 4;21(12):e3002416. doi: 10.1371/journal.pbio.3002416 (PMC10695390; doi:10.1371/journal.pbio.3002416)
Supplement: S1 Text — (DOCX) [file pbio.3002416.s008.docx]

# **S1 Text. Detailed discussion on phage CRISPRi results**

Here we present a detailed observation of CRISPRi assays. Discussion on CRISPRi polarity and how that adds ambiguity to gene essentiality is presented in the main text.

**Note A: CRISPRi knockdown assays of Lambda phage**

Phage infection cycle begins with adsorption of phages to the host cell receptors and ejection of phage DNA into host cytoplasm. For temperate phages such as λ, one of the early steps in the infection cycle is to commit either to lytic or lysogenic life cycle[[1]](https://paperpile.com/c/SHBZto/lJW3e). During this decision making step, proteins encoded from genes *cI*, *cII*, *cIII* and *cro* form a regulatory network that decide the cell fate depending upon factors such as environmental signals, number of infecting phages, to name a few[[2]](https://paperpile.com/c/SHBZto/ZEmZh). CI is the master lysogenic repressor and maintains lysogeny by preventing the expression of lytic genes[[1]](https://paperpile.com/c/SHBZto/lJW3e). Whereas, CII and CIII are required to initially activate the synthesis of CI[[3,4]](https://paperpile.com/c/SHBZto/c1XLb+6LKL).

Here we used lytic λcI857 phage for CRISPRi assays (Fig. 3). When *cI* or *cIII* was targeted in this study, the EOP of λ was ~1 suggesting that CI or CIII were not required for efficient propagation. The essentiality of *cII* could not be tested because of the lack of PAM site within the gene, but *cII* has been shown to be non-essential for plaque formation in earlier study[[5]](https://paperpile.com/c/SHBZto/TgVAY). As CIII and CI are required for establishing and maintaining lysogeny, it is understandable that their downregulation do not perturb the plating efficiency and are thus non-essential for phage propagation. These observations are also supported by normal plating phenotypes of these mutants in these genes in earlier studies[[4,6,7]](https://paperpile.com/c/SHBZto/6LKL+62BM+8mut).

During lysogeny, the λ genome is integrated into the host genome. Int plays a crucial role in lysogeny as Int facilitates the integration of the λ genome into the bacterial genome[[1,8]](https://paperpile.com/c/SHBZto/aoHt1+lJW3e). On the contrary, differential regulation of Int and Xis facilitate excision of the λ genome during the lytic switch[[9]](https://paperpile.com/c/SHBZto/ltCLA). The EOP was ~1 when either *int* or *xis* was targeted, which shows that neither Int nor Xis is required for a successful infection cycle.

Cro is the lytic repressor in λ and plays a major role during lysogenic state to lytic switch[[1]](https://paperpile.com/c/SHBZto/lJW3e). Cro reduces the transcript levels from P_L_ and P_R_[[10]](https://paperpile.com/c/SHBZto/pJBMr), thus reducing the expression of cII, which causes a switch to the lytic pathway[[1]](https://paperpile.com/c/SHBZto/lJW3e). When *cro* was repressed, the plating defect was severe and no individual plaques were seen, which suggests that Cro is essential for plaque formation. In the absence of Cro, lysogeny is stabilized or the lytic switch is repressed and thus, no plaques are seen on the lawn of host cells following phage infection. Similar observations were also made with *cro^-^* mutants in previous studies[[11]](https://paperpile.com/c/SHBZto/EEtu7).

Following the switch from lysogenic to lytic growth, early transcripts are generated from P_R_ and P_L_ promoters[[3]](https://paperpile.com/c/SHBZto/c1XLb). One of the genes expressed from P_L_ is *N*. N functions as an anti-terminator protein to allow expression of downstream genes on both P_L_ and P_R_ whose transcription is otherwise terminated at terminators tL1 and tR1[[3]](https://paperpile.com/c/SHBZto/c1XLb). CRISPRi targeting of *N* causes reduction of EOP by ~4 logs, which indicates that N is essential for efficient lytic growth. Our results are in agreement with prior study on essentiality of *N* in lytic growth[[12]](https://paperpile.com/c/SHBZto/0qg9u).

Following the expression of N, full length P_R_ (originating at ~38 kb and transcribing right) and P_L_ transcripts (originating at ~36 kb and transcribing left) are transcribed[[3]](https://paperpile.com/c/SHBZto/c1XLb). The transcript originating from P_R_ encompasses genes from *cro* to *Q*. This operon encodes genes involved in DNA replication, O and P[[13]](https://paperpile.com/c/SHBZto/l7518), and another antitermination protein, Q. Thus, knockdown of N also have pleiotropic effects on DNA replication[[14]](https://paperpile.com/c/SHBZto/hEaim). When we targeted CRISPRi to O or P, no plaques were observed, which indicates that both O and P are essential for plaque formation in agreement with previously established results[[15,16]](https://paperpile.com/c/SHBZto/r9e33+GZdb0) (see main text). Q, the terminal gene in the P_R_ operon, is involved in synthesis of late mRNAs and proteins[[14]](https://paperpile.com/c/SHBZto/hEaim). CRISPRi of Q reduced the EOP by ~5 logs, which shows that Q is also essential for efficient lytic replication.

This P_R_ operon also consists of other genes following P and preceding Q, in the following order *ren*, *ninB/C/D/E/F/G/H/I*. These genes have been described as non-essential for propagation[[3]](https://paperpile.com/c/SHBZto/c1XLb) of λ. However, *ren*, *ninB/C/F/G/I* genes appeared essential in our CRISPRi assays as knockdown of these genes caused severe defects in the EOP. These conflicting observations could be attributed to the CRISPRi polar effects (see, discussion in the main text), which suggests that non-essential genes in an operon that contains a downstream essential gene might appear as essential in this assay. Essentiality of *ninD/E/H* couldn’t be tested in this study owing to the lack of PAM site in these genes. To characterize the polarity effect on antiterminator *Q* knockdown and to validate if the lethality of *nin* knockdown can be rescued, we carried out CRISPRi assays on Nin region, genes *O*, *P* and *Q* in presence of plasmid pQ[[17]](https://paperpile.com/c/SHBZto/w3NkK) by overexpressing Q in *trans*. These results confirmed that the Nin region is non-essential for the lytic cycle of λ and the confounding CRISPRi data was because of polarity effect on essential antiterminator Q protein expression.

The P_L_ operon contains 20 genes, starting from N to ea47. This operon contains three regions of non-essential gene clusters[[3]](https://paperpile.com/c/SHBZto/c1XLb) : the first region extends from *ral* to *kil*, the second region extends from orf *60a* to *ea8.5* and the third region extends from *ea59* to *ea47*. Some of these genes provide biologically significant functions such as homologous recombination (*exo*, *bet,* *gam*)[[18]](https://paperpile.com/c/SHBZto/m2KUP), inhibition of host cell division (*kil*)[[19]](https://paperpile.com/c/SHBZto/ZruxM), yet their downregulation does not seem to have any effects on the EOP. Previous study has shown that lytic replication of recombination-deficient (*red*^-^) λ yields lower burst size[[20,21]](https://paperpile.com/c/SHBZto/4wVVc+fCbdH), however, the EOP was normal when each of the three red genes was targeted in our CRISPRi assays. The knockdown of *ral* doesn’t result in defect in the EOP because the host strain used in the assay doesn’t have functional type I restriction system[[22,23]](https://paperpile.com/c/SHBZto/fs6TR+EVPtN). We repeated *ral* knockdown assays in two indicator strains with active type I restriction system and found it to be essential. The *sieB* gene that is located in the opposite strand to the P_L_ operon is also not required for successful lytic replication in our assay conditions.

The assembly of virions requires several structural components such as capsid proteins, tail proteins and DNA packaging complexes. Besides there are other scaffolding or chaperone proteins that assist in the assembly of mature virions from the structural protein components[[24–27]](https://paperpile.com/c/SHBZto/jEyz1+AswiB+pohpT+IfV99). The protein components involved in the structural assembly of λ virions are transcribed from P_R’_ located at ~44.5 kb region of the genome (bottom right of the genome map, Fig. 3)[[3]](https://paperpile.com/c/SHBZto/c1XLb). These proteins are involved in capsid morphogenesis (Nu1, A, B, C, Nu3, D, E, FI, FII) or tail morphogenesis (V, G, G-T, H, M, L, K, I, J). All of these proteins have been shown previously to be required for stable or functional virion formation[[3,28–37]](https://paperpile.com/c/SHBZto/vgMEU+vp823+Yf4U4+2J23u+bgOit+Gnw2u+tdDOp+XuiIn+asG38+P4jtF+c1XLb). Our results are consistent with previously shown data and show that all of these genes are essential for lytic replication as CRISPRi knockdown of any one of these genes result in severe EOP defects (~5 logs) or no plaque formation (see Discussion on this in main text).

The ultimate step in a lytic phage replication is the lysis of the host cells to release the progeny virions. In gram-negative bacteria, this step involves degradation of the peptidoglycan layer and disruption of the inner and outer membrane[[38]](https://paperpile.com/c/SHBZto/q7o5i). In λ, host lysis function is carried out by four genes- *S*, *R*, *Rz* and *Rz1*[*[39]*](https://paperpile.com/c/SHBZto/KLcj9). The holin and antiholin of λ are the products of the same gene *S* and the two protein products, performing contradictory functions, are synthesized because of two translation start sites[[40]](https://paperpile.com/c/SHBZto/WlJit). S105 functions as holin, whereas S107 functions as anti-holin[[40]](https://paperpile.com/c/SHBZto/WlJit). Since S105 and S107 are in the same translational frame, similar effects are expected from any type of gene mutation. In our CRISPRi assays, we observed ~4-5 logs EOP defect, which indicates that the translation of *S* is essential for plaque formation. Earlier study has shown a delayed lysis time for λ *S* mutant[[40]](https://paperpile.com/c/SHBZto/WlJit). In our assay set up, it is not possible to dissect if the observed effects arise from repression of S105 or S107, but it can be hypothesized that the observed effects arise from effective repression of S105 as the followings steps of lysis pathway are blocked by the absence of holin. CRISPRi knockdown of endolysin R also did not result in significant plating defect. Disruption of Rz or Rz1 by introducing amber mutation in the respective genes resulted in ~2 logs EOP defect[[41]](https://paperpile.com/c/SHBZto/8PPg4). In our assays, CRISPRi of *Rz* resulted in EOP decrease, hence *Rz* appears as essential, and the plaques were small and turbid suggesting that the lysis of host cells was affected by knockdown of *Rz.* The effect of *Rz1* knockdown couldn’t be tested because of the lack of optimal PAM site.

The λ genome also contains other accessory genes that perform different functions such as *lom*, *bor*, *Ea47*, *Ea31*, *Ea59*, *rexA*, *rexB*[*[3]*](https://paperpile.com/c/SHBZto/c1XLb). CRISPRi knockdown of any of these genes did not alter the EOP significantly, thus indicating that these genes are also not essential for efficient lytic replication.

**Note B: CRISPRi knockdown assays of P1 phage**

One of the early proteins expressed from the P1 genome is Cre, which is a recombinase and helps in the circularization of the genome[[42]](https://paperpile.com/c/SHBZto/8o3TU). *cra*, upstream of *cre*, is also believed to play a role in circularization of the P1 genome[[43,44]](https://paperpile.com/c/SHBZto/dY14X+mVanJ). CRISPRi targeting of both *cra* or *cre* did not result in any EOP defect. Cre is also required for the segregation of P1 plasmids during lysogenic growth, but since the virulent mutant is used in this assay, it is not surprising that knockdown of *cre* doesn’t cause any EOP defect[[43]](https://paperpile.com/c/SHBZto/dY14X). Similarly, *c8* that is required for establishment of P1 lysogeny[[45]](https://paperpile.com/c/SHBZto/0fXdB) also appears non-essential in our CRISPRi assay. Two genes *ref* and *mat* are present downstream of *c8*. Transposon-insertion mutants of *ref* reported to show no growth defect in either lytic or lysogenic conditions[[46]](https://paperpile.com/c/SHBZto/QxeQX). We were able to recapitulate this observation in lytic growth conditions as the EOP appears normal when *ref* is targeted. Gene *mat* has been shown to be essential for virion production as amber mutation in *mat* results in production of particles with empty heads and unstable tails[[47]](https://paperpile.com/c/SHBZto/Bklnc), and severe plating defect of ~5 logs[[48]](https://paperpile.com/c/SHBZto/K5kFu). However, our CRISPRi assay was unable to replicate these findings as EOP appeared to be reduced by only ~1 log. It is possible the designed crRNA is inefficient in our assay.

In the negative strand of the genome encompassing 4kb to 16kb, P1 contains antirestriction and restriction-modification (R-M) components[[49]](https://paperpile.com/c/SHBZto/mnOgf). These genes *darB* and *ulx* form the antirestriction system to protect the P1 genome from Type I restriction-modification systems[[50,51]](https://paperpile.com/c/SHBZto/cyzMx+Ivb0t). These antirestriction systems are essential to protect the genome in host cells having active R-M systems, but in cells devoid of R-M systems, these components have been determined non-essential for efficient plaque formation[[50,51]](https://paperpile.com/c/SHBZto/cyzMx+Ivb0t). On the contrary, the same operon also contains genes *res* and *mod* that form a functional Type III R-M system that prevent P1 lysogens from inappropriately modified foreign DNA[[52]](https://paperpile.com/c/SHBZto/nP0T6). Mutants for both *res* or *mod* have been described earlier to show restriction or modification specific phenotypes[[52]](https://paperpile.com/c/SHBZto/nP0T6). In our CRISPRi assay as well, knockdown of either of these genes did not affect the EOP, suggesting that even though these genes might play important biological functions in some hosts, both are non-essential for efficient plaque formation in our assay system. This operon also contains gene *lxc*, the product of which is known to modulates the activity of C1 (lytic repressor) by enhancing binding of C1 to its operators[[53]](https://paperpile.com/c/SHBZto/DDR5L). In agreement with earlier data[[53]](https://paperpile.com/c/SHBZto/DDR5L), *lxc* also appeared non-essential in our CRISPRi assay.

The P1 genome has genes *prt* and *pro* in the ~16kb-19kb region. *prt* encodes the portal protein that is essential for DNA packaging into capsid and *pro* encodes putative head processing protease.These genes *prt* and *pro* were not mapped definitively in the studies that propose the respective functions of these proteins[[54–56]](https://paperpile.com/c/SHBZto/lYE0o+edA2h+euFLn). The genomic loci of these two genes were mapped when the P1 genome was sequenced[[49]](https://paperpile.com/c/SHBZto/mnOgf). In our CRISPRi assay, both genes appear essential for successful lytic replication, as knockdown of either gene resulted in no plaque formation.

The genome region of ~19-20 kb contains lysis genes *lydE*, *lydD* and *lyz*. *lyz* encodes for lysozyme that degrades the peptidoglycan layer during host cell lysis[[57]](https://paperpile.com/c/SHBZto/ZfS7u). *lyz*- P1 phages produce normal virions, but the host cells are not lyzed even when chloroform is added[[56]](https://paperpile.com/c/SHBZto/euFLn). In our assays, knockdown of *lyz* results in mild EOP defect (~2 logs), which suggest that *lyz* is required for optimum replication of P1 at laboratory conditions. The functions of *lydE* and *lydD* have been proposed to be antiholin and holin respectively[[49]](https://paperpile.com/c/SHBZto/mnOgf), but we could not find any studies validating their functions. Both *lydE* and *lydD* appear non-essential in our assay conditions as their knockdown did not result in any EOP defect.

The genomic region of ~20-23 kb contains genes *ssb* and other insertion element associated genes *isaA*, *insB*, *insA* and *isaB*[*[49]*](https://paperpile.com/c/SHBZto/mnOgf). None of these genes appear required for efficient P1 replication in our assays. *ssb* is weakly expressed during lytic growth only[[58]](https://paperpile.com/c/SHBZto/n7xQB). In *E. coli*, SSB is essential for DNA replication, recombination and repair, and it has been shown that P1 SSB can compensate for mutant *E. coli* SSB[[58]](https://paperpile.com/c/SHBZto/n7xQB). However, to our knowledge, it has not been shown whether *E. coli* SSB can complement P1 SSB.

The genomic region ~23-30 kb comprises an operon encoding genes responsible for lytic, morphogenesis and antirestriction functions[[49,50]](https://paperpile.com/c/SHBZto/mnOgf+cyzMx). The genes encoding holin, *lydA* and antiholin, *lydB* have been deleted in a previous study and have been described as non-essential[[57,59]](https://paperpile.com/c/SHBZto/Ezard+ZfS7u). In our CRISPRi assay, the knockdown of *lydA* did not result in plating defect, but that of *lydB* resulted in minor plating defect, suggesting that in laboratory conditions holins and antiholins are not required for efficient replication. Another gene *lydC,* which is also encoded in the same operon, is hypothesized to function as holin[[49]](https://paperpile.com/c/SHBZto/mnOgf). Similar to *lydA*, CRISPRi targeting of *lydC* also did not result in any plating defect in our assay. The genes *hdf*, *darA*, *ddrA* and *ddrB* are involved in antirestriction function and have been shown previously to be non-essential[[50]](https://paperpile.com/c/SHBZto/cyzMx). Our data is in agreement with these prior results. The terminal gene of the operon, *hxr*, is of unknown function. CRISPRi knockdown of *hxr* did not result in any plating defect as described in earlier study with clean deletion[[50]](https://paperpile.com/c/SHBZto/cyzMx).

The genomic region ~30-45 kb encodes several structural genes required for tail morphogenesis[[49]](https://paperpile.com/c/SHBZto/mnOgf). The positive strand of region ~30-33kb contains genes *cin*, *Sv’* and *U’*. Cin is a site-specific recombinase that results in the inversion of the C-segment. This C-segment inversion results in generation of two host variations of P1 virions with tail fibers differing in C-terminal domains[[60]](https://paperpile.com/c/SHBZto/W1ale). *cin* has been previously determined to be non-essential[[60]](https://paperpile.com/c/SHBZto/W1ale). Our data is in agreement with these earlier findings as knockdown of *cin* did not result in significant EOP defect. *Sv’* and *U’*, which are located downstream of *cin*, code for the alternative C-terminal regions of the P1 tail fiber[[49,61]](https://paperpile.com/c/SHBZto/mnOgf+56Q67). Since *Sv’* and *U’* constitute an alternative set of tail fibers, *Sv’* and *U’* are essential during infection of specific hosts only. In our assay conditions, both *Sv’* and *U’* appear non-essential as our host is *E. coli* K-12. The negative strand of the region ~33-45 kb contains genes *pmgC*, *tub*, *pmgB*, *sit*, *pmgA*, *bplA*, *16*, *R*, *S* and *U*. The functions of *pmgC*, *pmgB* and *pmgA* have not been determined. However, all appear essential as knockdown of *pmgC*, *pmg*B or *pmgA* resulted in extreme EOP defects. Other genes have defined roles in tail morphogenesis[[49]](https://paperpile.com/c/SHBZto/mnOgf) such as tail tube (*tub*), tapemeasure (*sit*), baseplate (*bplA*, *16*)[[47,62]](https://paperpile.com/c/SHBZto/74maG+Bklnc) and tail fiber (*R,* *S*, *U*)[[62]](https://paperpile.com/c/SHBZto/74maG). CRISPRi knockdowns of these genes in our assays also resulted in extreme EOP defects. These results suggest that all genes located in between *pmgC* and *U*, including both *pmgC* and *U* as well, are essential for P1 propagation.

Immediately upstream of this cluster of essential genes is an operon that encodes genes *simA*, *simB* and *simC* that function to prevent superinfection. Based on the phenotype, P1 that is defective in superinfection exclusion has been described earlier, but it is not clear which gene has been disrupted[[63]](https://paperpile.com/c/SHBZto/AMkBG). In our assay conditions, knockdown of *simA*, *simB* or *simC* did not result in any EOP defect, which suggests that neither of these genes are essential for a successful infection cycle.

The positive strand of the genomic region 47-52 kb contains a cluster of genes (*c4*, *icd*, *ant*, *kil*, *repL*, *rlfA*, *rlfB*) some of which are part of the immunity cluster of P1. All of the genes in this operon appear non-essential in our CRISPRi assay as the EOP of P1 appear normal when any of these genes are targeted. C4 RNA negatively regulates Icd and Ant1/2 proteins. Phenotypic null mutants of c4 have been described earlier, but have not been mapped into the P1 genome[[64]](https://paperpile.com/c/SHBZto/i8iUy). Likewise, P1 mutants of *icd*[*[65]*](https://paperpile.com/c/SHBZto/txZ0o) or *ant*[*[66]*](https://paperpile.com/c/SHBZto/F9Ajn) have also been isolated earlier. Insertion mutants of *kilA* or double deletion mutants of *kilA*-*repL* have also been isolated earlier[[66]](https://paperpile.com/c/SHBZto/F9Ajn) and both mutants were described to be defective in lytic replication[[66]](https://paperpile.com/c/SHBZto/F9Ajn), however CRISPRi knockdown of either *kilA* or *repL* doesn’t result in any EOP defect in this study. Hence, the lytic replication defective P1 mutants described earlier[[66]](https://paperpile.com/c/SHBZto/F9Ajn) could be due to the polar effects. The terminal two genes of this operon *rflA* and *rflA* have been hypothesized to be associated with lytic replication[[49]](https://paperpile.com/c/SHBZto/mnOgf), but both were found to be non-essential in this assay.

The negative strand of the genome region ~52-64 region contains 14 genes, some of which are essential genes encoding structural components of the P1 virion. The genes in the 5’ region of the operon, *upfC*, *upfB*, *ppfA*, *mlp* and *upfA* do not have any predicted functions[[49]](https://paperpile.com/c/SHBZto/mnOgf) and appear non-essential in this CRISPRi assay. The next three genes downstream to *upfA*: *repA*, *parA* and *parB* are involved in replication of P1 genome during lysogenic growth[[67,68]](https://paperpile.com/c/SHBZto/0IIHq+BQP5X). The mutations in these genes have not been described earlier, but these genes appear non-essential in our CRISPRi assay as their knockdown did not result in significant EOP defect. Immediately downstream of *parB* contain a set of five essential genes that are required for synthesis of structural components of P1 virions: *23*, *22*, *21*, *pmgG* and *bplB*. Amber mutants of genes *23*, *22* and *21* have been isolated earlier[[47]](https://paperpile.com/c/SHBZto/Bklnc) and these genes appear essential in our CRISPRi assays as well as P1 cannot form any plaques when these genes are downregulated. Gene *23* encodes the major capsid protein, gene *22* encodes the tail sheath protein and gene *21* encodes either base plate or tail tube protein[[47,49]](https://paperpile.com/c/SHBZto/Bklnc+mnOgf). When *pmgG* or *bplB* is targeted, P1 cannot form any plaques either, which shows that products of both *pmgG* or *bplB* are required for propagation of P1. PmgG does not have any assigned functions, whereas BplB is hypothesized to form baseplate structure[[49]](https://paperpile.com/c/SHBZto/mnOgf). The terminal gene in this operon, *pmgF*, is suggested to be required for morphogenesis[[49]](https://paperpile.com/c/SHBZto/mnOgf), but appears to be non-essential in our CRISPRi assay.

The genomic region ~64-73 kb contains eleven genes, all of which appear non-essential in our CRISPRi assays as knockdown of any of these genes did not result in plating defect. The first two genes of the operon of the genomic region ~64-68 kb, *uhr* and *hrdC*, do not have any predicted functions[[49]](https://paperpile.com/c/SHBZto/mnOgf). The downstream gene to *hrdC* is *dmt* in the previously published P1 genome[[49]](https://paperpile.com/c/SHBZto/mnOgf). Dam methylates[[69]](https://paperpile.com/c/SHBZto/DKxkc) adenine residues in the sequence 5’-GATC-3’. dam^-^ P1 has been found to be viable in early study[[69]](https://paperpile.com/c/SHBZto/DKxkc). In the genome of P1vir used in this study, *dmt* is split into two segments and have been annotated as *dmt-A* and *dmt-B*. knockdown of both segments of *dmt* did not result in any EOP defect, however it is difficult to ascertain if either of the split segments of dmt is functional. The negative strand of the genomic region ~68-69 contains two genes, *upl* and *plp*, that appear non-essential in this assay and do not have any demonstrated function[[49]](https://paperpile.com/c/SHBZto/mnOgf). Five genes are present in the positive strand of the genomic region ~69-73 kb, all of which can be knocked down without resulting in any EOP defect. Gene products of *tciA*, *tciB* and *tciC* are suggested to be involved in tellurite or colicin resistance or inhibition of cell division[[49]](https://paperpile.com/c/SHBZto/mnOgf). *ban* encodes for a DnaB homolog and has shown to be non-essential in earlier study[[70]](https://paperpile.com/c/SHBZto/ADUDi). *dbn* is present downstream of ban and does not have any predicted functions[[49]](https://paperpile.com/c/SHBZto/mnOgf).

The negative strand of the genomic region ~73-78 contains six genes, two of which appear non-essential. knockdown of gene *25* or *26* did not result in any EOP defects. *gp26* has been suggested to be involved in baseplate formation and phage progeny with amber mutation in gene *26* has been described as having contracted tails[[47]](https://paperpile.com/c/SHBZto/Bklnc), but we did not see any significant plating defect when the gene was repressed. Gp25 has been suggested to be important for tail stability and phage progeny with amber mutation in gene *25* have been found to have abnormal tails[[47]](https://paperpile.com/c/SHBZto/Bklnc), but we did not see any significant EOP defect when gene *25* was targeted. There are four genes present downstream of gene *25* and these four genes (*7*, *24*, *6*, *5*) appear essential for efficient phage propagation as knockdown of these genes result in significant EOP defect (Fig 4. and Table 2 ). Gene *7* is required for tail stability and amber mutation in gene *7* results in the formation of abnormal tail structures[[47]](https://paperpile.com/c/SHBZto/Bklnc). Gene *24* is required for either baseplate or tail stability and amber mutation in gene *24* results in polysheath structure[[47]](https://paperpile.com/c/SHBZto/Bklnc). Gene *6* is required for determination of tail length as amber mutation in gene *6* results in shorter tails[[47]](https://paperpile.com/c/SHBZto/Bklnc). Likewise, gene *5* is required for baseplate formation and amber mutation in gene *5* results in particles with contracted tails[[47]](https://paperpile.com/c/SHBZto/Bklnc). Knockdown of gene *24* or *6* results in an EOP defect of ~120 and ~204- fold respectively, hence both genes *24* and *6* are required for efficient propagation of P1. Similarly, when gene 7 or 5 is knocked down, individual plaques are not seen even when the undiluted phage stock is plated. Thus it appears that both genes *7* and *5* are essential for a successful P1 infection cycle.

The genome region ~78-89 kb region contains a cluster of genes on the positive strand and only one gene in the negative strand. The genes that are present in the positive strand are *pmgL*, *pmgM*, *pmgN*, *pmgO*, *pmgP*, *ppp*, *pmgQ*, *pmgR*, *pmgS*, *pap*, *pmgT*, *pmgU*, *pmgV*, *upfM*, *upfN*, *upfO*, *hot*, *humD*, *phd*, *doc*, *pdcA* and *pdcB*. Gene *lxr* is present in the genomic region ~86 kb (Fig. 4). Of the genes present in this cluster, essentiality of *upfM* and *pdcA* could not be tested because of lack of protospacer sequence. When *pmgN* is knocked down, the EOP of P1 is reduced by ~70 fold (Table 2), which suggests that *PmgN* is required for efficient propagation of P1. All other genes in this cluster that were knocked down did not result in any EOP defect, suggesting that those genes are not essential for P1 propagation. Our CRISPRi assays failed to identify *pmgR* as an essential gene in disagreement with a recent report[[71]](https://paperpile.com/c/SHBZto/a51uU) that showed *pmgR* deletion led to defective P1 virion formation. It is possible that CRISPRi knockdown of *pmgR* was not successful in our assays either because of the nonoptimal spacer we designed or because leaky expression of *pmgR* was sufficient to allow plaque formation. It is also possible that this phenotypic difference stems from the different P1 phage (P1CMclr100) variant used in earlier study. As observed in the prior report[[71]](https://paperpile.com/c/SHBZto/a51uU), the phenotype of the *pmgR* deletion mutant was the only one (among other essential *pmgA*, *pmgB*, *pmgC*, and *pmgG*) that could be rescued by direct complementation, we speculate that the P1 virion assembly process can tolerate wide range of *PmgR* expression levels.

Immediately downstream of *pdcB*, in the genomic region ~89-92 kb, lies three genes: *lpa*, *pacA* and *pacB* (Fig. 4). P1 does not form plaques when any of these three genes are targeted, suggesting all three genes are essential for P1 propagation. *lpa* encodes a protein that is required for activation of the P1 late promoters[[72]](https://paperpile.com/c/SHBZto/IZ4lu) and it has been demonstrated that mutation in *lpa* results in morphogenesis defect[[47,56]](https://paperpile.com/c/SHBZto/euFLn+Bklnc). Gene products of *pacA* and *pacB* form a DNA packaging complex that cleaves P1 DNA at specific sites and packages DNA into procapsids during virion morphogenesis[[73]](https://paperpile.com/c/SHBZto/pT0bp).

**References**

1. [Court DL, Oppenheim AB, Adhya SL. A new look at bacteriophage lambda genetic networks. J Bacteriol. 2007;189: 298–304.](http://paperpile.com/b/SHBZto/lJW3e)

2. [Zeng L, Skinner SO, Zong C, Sippy J, Feiss M, Golding I. Decision making at a subcellular level determines the outcome of bacteriophage infection. Cell. 2010;141: 682–691.](http://paperpile.com/b/SHBZto/ZEmZh)

3. [Casjens SR, Hendrix RW. Bacteriophage lambda: Early pioneer and still relevant. Virology. 2015;479-480: 310–330.](http://paperpile.com/b/SHBZto/c1XLb)

4. [Kaiser AD. Mutations in a temperate bacteriophage affecting its ability to lysogenize Escherichia coli. Virology. 1957;3: 42–61.](http://paperpile.com/b/SHBZto/6LKL)

5. [Thomas R, Bertani LE. On the Control of the Replication of Temperate Bacteriophages Superinfecting Immune Hosts. Virology. 1964;24: 241–253.](http://paperpile.com/b/SHBZto/TgVAY)

6. [Lieb M. Mapping missense and nonsense mutation in gene cI of bacteriophage lambda: marker effects. Mol Gen Genet. 1976;146: 285–290.](http://paperpile.com/b/SHBZto/62BM)

7. [Truitt CL, Chu H, Walker JR. Bacteriophage lambda mutants (lambdatp) that overproduce repressor. J Virol. 1978;28: 877–884.](http://paperpile.com/b/SHBZto/8mut)

8. [Zissler J. Integration-negative (int) mutants of phage lambda. Virology. 1967;31: 189.](http://paperpile.com/b/SHBZto/aoHt1)

9. [Miller HI, Abraham J, Benedik M, Campbell A, Court D, Echols H, et al. Regulation of the integration-excision reaction by bacteriophage lambda. Cold Spring Harb Symp Quant Biol. 1981;45 Pt 1: 439–445.](http://paperpile.com/b/SHBZto/ltCLA)

10. [Svenningsen SL, Costantino N, Court DL, Adhya S. On the role of Cro in lambda prophage induction. Proc Natl Acad Sci U S A. 2005;102: 4465–4469.](http://paperpile.com/b/SHBZto/pJBMr)

11. [Eisen H, Brachet P, Pereira da Silva L, Jacob F. Regulation of repressor expression in lambda. Proc Natl Acad Sci U S A. 1970;66: 855–862.](http://paperpile.com/b/SHBZto/EEtu7)

12. [Galland P, Cortini R, Calef E. Control of gene expression in bacteriophage lambda: suppression of N mutants by mutations of the antirepressor. Mol Gen Genet. 1975;142: 155–170.](http://paperpile.com/b/SHBZto/0qg9u)

13. [Zylicz M, Liberek K, Wawrzynow A, Georgopoulos C. Formation of the preprimosome protects lambda O from RNA transcription-dependent proteolysis by ClpP/ClpX. Proc Natl Acad Sci U S A. 1998;95: 15259–15263.](http://paperpile.com/b/SHBZto/l7518)

14. [Court D, Sato K. Studies of novel transducing variants of lambda: dispensability of genes N and Q. Virology. 1969;39: 348–352.](http://paperpile.com/b/SHBZto/hEaim)

15. [Kleckner N. Amber mutants in the O gene of bacteriophage lambda are not efficiently complemented in the absence of phage N function. Virology. 1977;79: 174–182.](http://paperpile.com/b/SHBZto/r9e33)

16. [Mukai T, Ohkubo H, Shimada K, Takagi Y. Isolation and characterization of a plaque-forming lambda bacteriophage carrying a ColE1 plasmid. J Bacteriol. 1978;135: 171–177.](http://paperpile.com/b/SHBZto/GZdb0)

17. [Gründling A, Manson MD, Young R. Holins kill without warning. Proc Natl Acad Sci U S A. 2001;98: 9348–9352.](http://paperpile.com/b/SHBZto/w3NkK)

18. [Caldwell BJ, Bell CE. Structure and mechanism of the Red recombination system of bacteriophage lambda. Prog Biophys Mol Biol. 2019;147: 33–46.](http://paperpile.com/b/SHBZto/m2KUP)

19. [Haeusser DP, Hoashi M, Weaver A, Brown N, Pan J, Sawitzke JA, et al. The Kil peptide of bacteriophage lambda blocks Escherichia coli cytokinesis via ZipA-dependent inhibition of FtsZ assembly. PLoS Genet. 2014;10: e1004217.](http://paperpile.com/b/SHBZto/ZruxM)

20. [Signer ER, Weil J. Recombination in bacteriophage lambda. I. Mutants deficient in general recombination. J Mol Biol. 1968;34: 261–271.](http://paperpile.com/b/SHBZto/4wVVc)

21. [Enquist LW, Skalka A. Replication of bacteriophage lambda DNA dependent on the function of host and viral genes. I. Interaction of red, gam and rec. J Mol Biol. 1973;75: 185–212.](http://paperpile.com/b/SHBZto/fCbdH)

22. [Zabeau M, Friedman S, Van Montagu M, Schell J. The ral gene of phage lambda. I. Identification of a non-essential gene that modulates restriction and modification in E. coli. Mol Gen Genet. 1980;179: 63–73.](http://paperpile.com/b/SHBZto/fs6TR)

23. [Debrouwere L, Zabeau M, Van Montagu M, Schell J. The ral gene of phage lambda. II. Isolation and characterization of ral deficient mutants. Mol Gen Genet. 1980;179: 75–80.](http://paperpile.com/b/SHBZto/EVPtN)

24. [Leiman PG, Kanamaru S, Mesyanzhinov VV, Arisaka F, Rossmann MG. Structure and morphogenesis of bacteriophage T4. Cell Mol Life Sci. 2003;60: 2356–2370.](http://paperpile.com/b/SHBZto/jEyz1)

25. [Aksyuk AA, Rossmann MG. Bacteriophage assembly. Viruses. 2011;3: 172–203.](http://paperpile.com/b/SHBZto/AswiB)

26. [King J, Lenk EV, Botstein D. Mechanism of head assembly and DNA encapsulation in *Salmonella* phage P22. II. Morphogenetic pathway. J Mol Biol. 1973;80: 697–731.](http://paperpile.com/b/SHBZto/pohpT)

27. [Dokland T. Scaffolding proteins and their role in viral assembly. Cell Mol Life Sci. 1999;56: 580–603.](http://paperpile.com/b/SHBZto/IfV99)

28. [Weisberg RA, Sternberg N, Gallay E. The nu1 gene of coliphage lambda. Virology. 1979;95: 99–106.](http://paperpile.com/b/SHBZto/vgMEU)

29. [Harrison DP, Brown DT, Bode VC. The lambda head-tail joining reaction: purification, properties and structure of biologically active heads and tails. J Mol Biol. 1973;79: 437–449.](http://paperpile.com/b/SHBZto/vp823)

30. [Boklage CE, Wong EC, Bode VC. Functional abnormality of lambda phage particles from complemented FII-mutant lysates. Virology. 1974;61: 22–28.](http://paperpile.com/b/SHBZto/Yf4U4)

31. [Kaiser D, Syvanen M, Masuda T. Processing and assembly of the head of bacteriophage lambda. J Supramol Struct. 1974;2: 318–328.](http://paperpile.com/b/SHBZto/2J23u)

32. [Medina E, Wieczorek D, Medina EM, Yang Q, Feiss M, Catalano CE. Assembly and maturation of the bacteriophage lambda procapsid: gpC is the viral protease. J Mol Biol. 2010;401: 813–830.](http://paperpile.com/b/SHBZto/bgOit)

33. [Ray P, Murialdo H. The role of gene Nu3 in bacteriophage lambda head morphogenesis. Virology. 1975;64: 247–263.](http://paperpile.com/b/SHBZto/Gnw2u)

34. [Hohn B, Hohn T. Activity of empty, headlike particles for packaging of DNA of bacteriophage lambda in vitro. Proc Natl Acad Sci U S A. 1974;71: 2372–2376.](http://paperpile.com/b/SHBZto/tdDOp)

35. [Hendrix RW. Lambda II. Cold Spring Harbor Laboratory; 1983.](http://paperpile.com/b/SHBZto/XuiIn)

36. [Kemp CL, Howatson AF, Siminovitch L. Electron microscopy studies of mutants of lambada bacteriophage. I. General description and quantitation of viral products. Virology. 1968;36: 490–502.](http://paperpile.com/b/SHBZto/asG38)

37. [Szybalski EH, Szybalski W. A comprehensive molecular map of bacteriophage lambda. Gene. 1979;7: 217–270.](http://paperpile.com/b/SHBZto/P4jtF)

38. [Young R. Phage lysis: three steps, three choices, one outcome. J Microbiol. 2014;52: 243–258.](http://paperpile.com/b/SHBZto/q7o5i)

39. [Berry J, Summer EJ, Struck DK, Young R. The final step in the phage infection cycle: the Rz and Rz1 lysis proteins link the inner and outer membranes. Mol Microbiol. 2008;70: 341–351.](http://paperpile.com/b/SHBZto/KLcj9)

40. [Chang CY, Nam K, Young R. S gene expression and the timing of lysis by bacteriophage lambda. J Bacteriol. 1995;177: 3283–3294.](http://paperpile.com/b/SHBZto/WlJit)

41. [Zhang N, Young R. Complementation and characterization of the nested Rz and Rz1 reading frames in the genome of bacteriophage lambda. Mol Gen Genet. 1999;262: 659–667.](http://paperpile.com/b/SHBZto/8PPg4)

42. [Hochman L, Segev N, Sternberg N, Cohen G. Site-specific recombinational circularization of bacteriophage P1 DNA. Virology. 1983;131: 11–17.](http://paperpile.com/b/SHBZto/8o3TU)

43. [Sternberg N, Sauer B, Hoess R, Abremski K. Bacteriophage P1 cre gene and its regulatory region. Evidence for multiple promoters and for regulation by DNA methylation. J Mol Biol. 1986;187: 197–212.](http://paperpile.com/b/SHBZto/dY14X)

44. [Yarmolinsky MB, Sternberg N. Bacteriophage P1. In: Calendar R, editor. The Bacteriophages. Boston, MA: Springer US; 1988. pp. 291–438.](http://paperpile.com/b/SHBZto/mVanJ)

45. [Scott JR, Kropf MM. Location of new clear plaque genes on the P1 map. Virology. 1977;82: 362–368.](http://paperpile.com/b/SHBZto/0fXdB)

46. [Lu SD, Lu D, Gottesman M. Stimulation of IS1 excision by bacteriophage P1 ref function. J Bacteriol. 1989;171: 3427–3432.](http://paperpile.com/b/SHBZto/QxeQX)

47. [Walker JT, Walker DH Jr. Coliphage P1 morphogenesis: analysis of mutants by electron microscopy. J Virol. 1983;45: 1118–1139.](http://paperpile.com/b/SHBZto/Bklnc)

48. [Lehnherr H, Jensen CD, Stenholm AR, Dueholm A. Dual regulatory control of a particle maturation function of bacteriophage P1. J Bacteriol. 2001;183: 4105–4109.](http://paperpile.com/b/SHBZto/K5kFu)

49. [Lobocka MB, Rose DJ, Plunkett G 3rd, Rusin M, Samojedny A, Lehnherr H, et al. Genome of bacteriophage P1. J Bacteriol. 2004;186: 7032–7068.](http://paperpile.com/b/SHBZto/mnOgf)

50. [Piya D, Vara L, Russell WK, Young R, Gill JJ. The multicomponent antirestriction system of phage P1 is linked to capsid morphogenesis. Mol Microbiol. 2017;105: 399–412.](http://paperpile.com/b/SHBZto/cyzMx)

51. [Iida S, Streiff MB, Bickle TA, Arber W. Two DNA antirestriction systems of bacteriophage P1, *darA*, and *darB*: characterization of *darA*^-^ phages. Virology. 1987;157: 156–166.](http://paperpile.com/b/SHBZto/Ivb0t)

52. [Iida S, Meyer J, Bachi B, Stalhammar-Carlemalm M, Schrickel S, Bickle TA, et al. DNA restriction--modification genes of phage P1 and plasmid p15B. Structure and in vitro transcription. J Mol Biol. 1983;165: 1–18.](http://paperpile.com/b/SHBZto/nP0T6)

53. [Schaefer TS, Hays JB. The bof gene of bacteriophage P1: DNA sequence and evidence for roles in regulation of phage c1 and ref genes. J Bacteriol. 1990;172: 3269–3277.](http://paperpile.com/b/SHBZto/DDR5L)

54. [Razza JB, Watkins CA, Scott JR. Phage P1 temperature-sensitive mutants with defects in the lytic pathway. Virology. 1980;105: 52–59.](http://paperpile.com/b/SHBZto/lYE0o)

55. [Walker DH Jr, Walker JT. Genetic studies of coliphage P1. III. Extended genetic map. J Virol. 1976;20: 177–187.](http://paperpile.com/b/SHBZto/edA2h)

56. [Walker JT, Walker DH. Mutations in coliphage p1 affecting host cell lysis. J Virol. 1980;35: 519–530.](http://paperpile.com/b/SHBZto/euFLn)

57. [Schmidt C, Velleman M, Arber W. Three functions of bacteriophage P1 involved in cell lysis. J Bacteriol. 1996;178: 1099–1104.](http://paperpile.com/b/SHBZto/ZfS7u)

58. [Lehnherr H, Bendtsen JD, Preuss F, Ilyina TV. Identification and characterization of the single-stranded DNA-binding protein of bacteriophage P1. J Bacteriol. 1999;181: 6463–6468.](http://paperpile.com/b/SHBZto/n7xQB)

59. [Iida S, Arber W. Plaque forming specialized transducing phage P1: isolation of P1CmSmSu, a precursor of P1Cm. Mol Gen Genet. 1977;153: 259–269.](http://paperpile.com/b/SHBZto/Ezard)

60. [Iida S, Meyer J, Kennedy KE, Arber W. A site-specific, conservative recombination system carried by bacteriophage P1. Mapping the recombinase gene cin and the cross-over sites cix for the inversion of the C segment. EMBO J. 1982;1: 1445–1453.](http://paperpile.com/b/SHBZto/W1ale)

61. [Iida S. Bacteriophage P1 carries two related sets of genes determining its host range in the invertible C segment of its genome. Virology. 1984;134: 421–434.](http://paperpile.com/b/SHBZto/56Q67)

62. [Guidolin A, Zingg JM, Arber W. Organization of the bacteriophage P1 tail-fibre operon. Gene. 1989;76: 239–243.](http://paperpile.com/b/SHBZto/74maG)

63. [Devlin BH, Baumstark BR, Scott JR. Superimmunity: characterization of a new gene in the immunity region of P1. Virology. 1982;120: 360–375.](http://paperpile.com/b/SHBZto/AMkBG)

64. [Baumstark BR, Scott JR. The c4 gene of phage P1. Virology. 1987;156: 197–203.](http://paperpile.com/b/SHBZto/i8iUy)

65. [Heinrich J, Citron M, Gunther A, Schuster H. Second-site suppressors of the bacteriophage P1 virs mutant reveal the interdependence of the c4, icd, and ant genes in the P1 immI operon. J Bacteriol. 1994;176: 4931–4936.](http://paperpile.com/b/SHBZto/txZ0o)

66. [Hansen EB. Structure and regulation of the lytic replicon of phage P1. J Mol Biol. 1989;207: 135–149.](http://paperpile.com/b/SHBZto/F9Ajn)

67. [Chattoraj DK, Snyder KM, Abeles AL. P1 plasmid replication: multiple functions of RepA protein at the origin. Proc Natl Acad Sci U S A. 1985;82: 2588–2592.](http://paperpile.com/b/SHBZto/0IIHq)

68. [Davis MA, Martin KA, Austin SJ. Biochemical activities of the parA partition protein of the P1 plasmid. Mol Microbiol. 1992;6: 1141–1147.](http://paperpile.com/b/SHBZto/BQP5X)

69. [Coulby JN, Sternberg NL. Characterization of the phage P1 dam gene. Gene. 1988;74: 191.](http://paperpile.com/b/SHBZto/DKxkc)

70. [D’Ari R, Jaffe-Brachet A, Touati-Schwartz D, Yarmolinsky MB. A dnaB analog specified by bacteriophage P1. J Mol Biol. 1975;94: 341–366.](http://paperpile.com/b/SHBZto/ADUDi)

71. [Gonzales MF, Piya DK, Koehler B, Zhang K, Yu Z, Zeng L, et al. New Insights into the Structure and Assembly of Bacteriophage P1. Viruses. 2022;14. doi:](http://paperpile.com/b/SHBZto/a51uU)[10.3390/v14040678](http://dx.doi.org/10.3390/v14040678)

72. [Lehnherr H, Guidolin A, Arber W. Bacteriophage P1 gene 10 encodes a trans-activating factor required for late gene expression. J Bacteriol. 1991;173: 6438–6445.](http://paperpile.com/b/SHBZto/IZ4lu)

73. [Skorupski K, Pierce JC, Sauer B, Sternberg N. Bacteriophage P1 genes involved in the recognition and cleavage of the phage packaging site (pac). J Mol Biol. 1992;223: 977–989.](http://paperpile.com/b/SHBZto/pT0bp)
