# Supplementary material for: Systematic and scalable genome-wide essentiality mapping to identify nonessential genes in phages
Source: PLoS Biol. 2023 Dec 4;21(12):e3002416. doi: 10.1371/journal.pbio.3002416 (PMC10695390; doi:10.1371/journal.pbio.3002416)
Supplement: Attachment — Submitted filename: Response to Reviewers.docx [file pbio.3002416.s010.docx]

**Response to Reviewers (in Blue)**

**Reviewer #1:**

*This paper by Piya et al. primarily describes the deployment of CRISPRi via dCas12a to globally assess gene essentiality within two model bacteriophage. Using their identification of non-essential regions of the phage genomes in a practical example they then place genetic barcodes into phage lambda and P1 genomes and show how these can be used to enumerate and track phage populations within a mixed culture.*

*Overall, the paper is very clearly written and the logic and reasoning behind the experiments is well explained. The work is important and would be expected to be broadly applicable across at least the dsDNA phages, and likely ssDNA phages as well (although no example is given) that infect E. coli and closely related species. The plasmids as they are currently configured may not function in more distant host groups, but the principle outlined in this work should still hold if porting over to different bacterial families. The materials in the work are very well described and using the plasmids with different CRISPRi oligos would be straightforward. If the authors have not already, I encourage them to deposit the plasmids with Addgene so they are easily accessible.*

We would like to thank the reviewer for their kind words and comments on our work. We are preparing the vector backbone/samples to deposit them at Addgene.

*Figures: I'm not sure if this is due to the journal's reformatting of PDFs, but most of the figures show as fuzzy and some text is illegible (e.g. Fig 1)*

Probably this was caused by the journal's formating/conversion process. We have revised the text within all figures and uploaded all figures as high resolution images with this revised submission.

*Both phage lambda and P1 are temperate phage and I was wondering as I read the paper how you were dealing with that possibility. I see it explained in the Materials and Methods, but it would be good to mention in the main text that variants being used in the work are lysogen-deficient.*

We agree with the reviewer. We have edited the main text to address this and included this following line:

Line: 104

“We first benchmark the CRISPRi technology by applying it to a known set of essential and non-essential genes in both phages (lytic variants, λcI857 and P1vir, here onwards as lambda or λ and P1 respectively), and then extend it genome-wide to query essentiality of all genes in both phages.“

*The non-targeting crRNA was not fully described in the text. For example, what does it target? Is it similar GC-content to other oligos used?*

The non-targeting crRNA targets P1 phage in lambda CRISPRi assays while it targets lambda phage in P1 CRISPRi assays. The point was that it is a valid binding partner for the dCas12a protein while not expected to have any target in either phage or host genome.

We have incorporated this point in the methods section as below:

Line: 509

“The non-targeting crRNA targets P1 phage gene *23* in lambda CRISPRi assays while it targets lambda phage gene *E* in P1 CRISPRi assays.”

*The pros and cons of using dCas12a versus dCas9 are not discussed sufficiently. Why was dCas12a chosen? Would you expect similar results with dCas9? Why or why not?*

Thanks for this feedback. We have incorporated this following text to address this point. We hope that these additions to the text sufficiently capture the rationale for using dCas12a.

In the introduction, we have added

Line: 86

“While CRISPRi was first developed using dCas9, alternative Cas variants like dCas12a have achieved efficient knockdown in diverse bacteria (PMIDs: 34298016; 29980561;32379967). Both dCas9 and dCas12a are similarly effective for CRISPRi in many circumstances; however, advantages of dCas12 include more efficient restriction of a covalently modified phage genome (for example, T4 phage PMID: 32938767) and simpler cloning of dual crRNAs on short oligos relative to longer dCas9 single guide RNAs.”

In the discussion, we noted

Line: 384

“Furthermore, the simple multiplexability of dCas12a crRNAs (for example, dual crRNAs targeting two genes) could enable rapid, systematic investigation of synthetic lethal phage gene pairs.”

A major advantage of dCas12a is that it processes its own CRISPR array, so dual crRNAs targeting two genes simultaneously can be seamlessly designed, cloned, and tested.

As systematic and extended comparison of different Cas systems for targeting phage genomes is not the focus of this work and thus distracting from the main findings, here we share our unpublished data for reviewers in good faith. The following figure shows efficiency of knocking down genomically encoded RFP expression with several dCas variants. Essentially, this data indicates that dLbCas12a (E925Q mutant of *Lachnospiraceae* bacterium ND 2006) and dAsCas12a (E908Q mutant of *Acidomonococcus* sp. BV3L6) are as effective or better than CRISPRi with prototypical dSpyCas9, consistent with literature we have cited in which dCas12a performs CRISPRi well in prokaryotes. We are still investigating the mechanism behind this variability in efficiency.

*Amber mutants are treated as "ground truth" in this work, but have the authors thought about whether some amber mutants may also be polar mutations? Translation is thought to protect transcripts from degradation and if translation is stopped over a large section of mRNA this may destabilize the transcript causing a polar effect. One older article seems to show this may be possible (*[*https://doi.org/10.1016/0022-2836(68)90144-7*](https://doi.org/10.1016/0022-2836(68)90144-7)*) although the phage is very different that lambda or P1.*

We agree that amber mutants do not always represent clean gene deletions. However, for lambda, amber mutants are not polar because of the antitermination activity of N and Q. This point has been demonstrated unambiguously before, for example PMID 5475574; 325882; 4601822; 6217346; 2526335.

Compared to lambda, there is no direct experimental evidence of a protein playing a role of antitermination in phage P1. Nevertheless, there are strong indications that P1 does encode antiterminators (PMID 3170487; 15489417) and we speculate that like lambda, these antiterminators may overcome polarity of amber mutations.

Nevertheless, we agree that how amber mutations impact translation of downstream coding sequence by regulating differential accessibility of ribosomes to ribosome binding sites (especially in longer transcripts) in dsDNA phages is not systematically characterized. These PMID 5475574; 325882; 4601822; 6217346; 2526335 references indicate that antiterminator proteins appear to be sufficient to suppress amber mutant polarity.

*Supplementary is misspelled in several different parts of the manuscript*

Thank you. We have corrected the spelling errors.

*Figure 4: the color choice for essential (red) and intermediate phenotype (purple) is not discernible in the figures. Need to change colors so they have more contrast. It would also be very helpful to harmonize the colors between Fig 3, 4 and Tables 1 and 2.*

Thank you. We have addressed this point. We have revised figures, but kept the Tables 1 and 2 clean for easy readability.

*Table 2: The EOP average column has a large number of instances of "essential" instead of a quantitative measure, which is confusing.*

Thank you. We have addressed this point.

*The "Downstream application" section is not introduced with sufficient detail to really understand what is going on with the experiments. For example, how long are the phage cultured before measuring? This is pertinent because small fitness defects would be expected to have a detectable effect over large numbers of generations. Fig 5C it is impossible to differentiate the symbols.*

Thanks for the feedback. We have revised and improved the visualization of Fig 5C. The barcoded phages were mixed in different ratios and incubated at room temperature before subjecting them to PCR for amplifying the Barcode. We have addressed this by providing more clarity on this work

Line: 250

“As a proof of concept, we inserted a unique DNA barcode in genes *res* (between *cra*-*darB*) and *red*, of P1 and lambda respectively. We used a homologous recombination approach followed by nuclease active Cas12a-based counter selection for barcoded phages in a two-step process (Methods). Successful DNA barcode insertions into phage genomes were then confirmed by Sanger sequencing of the insertion locus. With these two *b*c (barcoded) constructs, we tested whether we could quantify different phage combinations. To do this, we mixed phage P1-bc and λ-bc in different ratios, incubated at room temperature for 30 minutes and subjected them to Barseq PCR sequencing[^69,70^](https://paperpile.com/c/BUmUl7/r6Rco+oT5cf). Our Barseq quantification method not only successfully quantified different ratios of barcoded phage P1 and lambda, but also captured the differences in plaque-forming units/ml to barcode abundance”

**Reviewer #2:**

*The manuscript by Piya et al. from the Arkin and Mutalik labs reports the application of CRISPRi to bacteriophages using a setup based on catalytically inactive Cas12a. In difference to previous work using similar technology, the authors target (almost) every gene in two phages and thus report on the first true genome-wide CRISPRi screens in bacterial viruses. This is certainly a breakthrough for the field, but in the current form constitutes rather quantitative than qualitative progress compared to previous work.*

We would like to thank the reviewer for their kind words and comments on our work.

*The paper is very well written and structured comprehensibly. The methodology is described very nicely with considerable detail which would facilitate wide application of the authors' technology.*

Thank you.

*The introduction appropriately introduces the great need for genome-wide screens in bacteriophages to probe the biological functions of the vast amount of phage genes of unknown function. However, it is not optimal that CRISPR-Cas12a and its use for CRISPRi are just dropped onto the non-expert reader without further explanations. I would therefore suggest to (at least very shortly) introduce the key differences between CRISPR-Cas9 (that many readers have likely heard about) and CRISPR-Cas12a to explain why the authors chose the latter and not the former, even though CRISPR-Cas9 is by far most often used for microbial CRISPRi.*

We thank the reviewer for raising this point. A similar point was raised by Reviewer 1.

We have addressed this comment as described above for Reviewer 1, pasting here for clarity:

We have edited the abstract and removed dCas12 to improve the readability. Now the edited line reads as

Line:40

“As proof of concept, we use an arrayed genome-wide CRISPR interference (CRISPRi) assay to map gene essentiality landscape in the canonical coliphages λ and P1.”

In the introduction, we have added Line: 86

“While CRISPRi was first developed using dCas9, alternative Cas variants like dCas12a have achieved efficient knockdown in diverse bacteria. Both dCas9 and dCas12a are similarly effective for CRISPRi in many circumstances; however, advantages of dCas12 include more efficient restriction of a covalently modified phage genome (T4) and simpler cloning of dual crRNAs on short oligos relative to longer dCas9 single guide RNA.,”

In the discussion, we noted, Line: 384

“Furthermore, the simple multiplexability of dCas12a crRNAs could enable rapid, systematic investigation of synthetic lethal phage gene pairs.” A major advantage of dCas12a is that it processes its own CRISPR array, so dual crRNAs targeting two genes simultaneously can be seamlessly designed, cloned, and tested.

We hope that these additions to the text sufficiently capture the rationale for using dCas12a.

*Furthermore, I feel that previous use of genome-wide CRISPRi to study viral biology needs to be referenced (Hein and Weissman, Nature Biotechnology (2021)) and explained with view to the question why the same approach could not be simply applied to bacteriophages.*

We have included this reference in our revised manuscript. This previous work by Hein and Weissman, Nature Biotechnology (2021) used pooled gRNA for studying *human cytomegalovirus* infection pathways and gene essentiality in human cells. We agree with the reviewer that the pooled fitness assays as used in Heln and Weissman (2021) can be applied to phages (see below about a new preprint).

We have used similar pooled fitness assays in bacteria to study the role of bacterial genes in antibiotic and metal tolerance and also to study the molecular mechanisms of phage resistance. As there are no prior studies on using CRISPRi technology for phage gene-essentiality mapping at genome-wide scale, and to establish the methodology and to avoid potential “jackpot effects” (enrichment of stronger phenotypes at the cost of weaker and intermediate fitness traits) commonly seen in pooled fitness assays, we carried out systematic single crRNA CRISPRi assay in an arrayed format. Because of the arrayed assay format, we could uncover potential polarity issues with high confidence and design experiments to overcome some of them (for example, expressing Q in *trans* while targeting Nin region). We believe that our foundational work as presented here will caution other researchers while using CRISPRi technology for phage gene essentiality mapping to look out for polarity effects while interpreting the data.

Now, we and others in the field can extend this technology for other phage-host systems and in different assay formats including pooled assays. For example, while this manuscript was under review, successful implementation of dCas13 based genome-wide pooled CRISPRi screen was reported for diverse phages[(https://www.biorxiv.org/content/10.1101/2023.09.18.558157v1)](https://paperpile.com/c/Bu0SE4/zUk4) and point to a rich future of diverse functional genomics tools to study phage biology.

We have included this reference in the discussion section as

Line: 373

“While this manuscript was under review, successful implementation of dCas13 based genome-wide pooled CRISPRi screen was reported for diverse phages[[80]](https://paperpile.com/c/Bu0SE4/zUk4) and point to a rich future of diverse functional genomics tools to study phage biology.”

*The results section begins with a nicely comprehensible and detailed description of how the authors set up CRISPRi using dCas12a. The authors' validation of the system's basic functionality to distinguish between essential and non-essential genes is very convincing.*

Thank you.

*However, in my view the quantification of knockdown phenotypes in figure 1 is not optimal, though I acknowledge that this is a never-ending discussion in the field. The authors write about a 10'5- to 10'6fold reduction in plating efficiency (line 126). However, there is no plaque formation visible for the knockdowns of essential genes, only "lysis from without". I therefore find it misleading to talk about a reduction in plating because no plating (i.e., plaque formation) can be seen.*

We have edited the main text to address this point. The next text reads as follows:

Line:132

“We observed that induction of CRISPRi targeting essential genes *E* and *nu1* of lambda and *mcp*, *pacA* and *sit* of P1 all showed severe compromise in phage growth (measured as plaque formation), whereas targeting nonessential genes *ppp*, *upfB* or *ddrB* of P1 did not.”

*Subsequently, the authors use their CRISPRi approach to query (near-)genome-wide gene essentiality in phages lambda and P1 gene by gene. These phages were chosen because generations of researchers have already generated a considerable body of work on gene essentiality for these phages which provides a very convincing benchmark for the authors' technology. The results are indeed largely in line with expectations from previous literature. However, the authors also observe both polar effects and consequences of incomplete knockdowns. These are known downsides of CRISPRi per se and thus do not constitute a specific problem of the authors' approach, even though they later discuss (but do not try) overcoming at least some of the polar effects by using catalytically inactive Cas13a instead of Cas12a.*

We agree with the reviewer’s comment that the polarity issue of CRISPRi is a difficult one to overcome. As a recent work highlights, the issue of polarity is true even for classical methods (PMID: 34491080).

Even with dCas13 which targets mRNA compared to DNA targeting dCas12, we will see some of these effects (probably less than dCas12 or dCas9, unpublished data from our lab, but also shown here <https://www.biorxiv.org/content/10.1101/2023.10.11.561958v1> and <https://www.biorxiv.org/content/10.1101/2023.09.18.558157v1>) as translation and transcription processes are coupled. The dCas13 knockdown effect on the target region of mRNA will also impact the transcript stability and translation process of the entire transcript in addition to the transcription of that transcript. Like a number of bacterial CRISPRi studies highlighting the polarity issues, we will have to be extra careful in assigning phage genes as “essential”.

*At the end, the authors inserted barcodes at different loci of their two model phages that their knockdown approach had identified or validated as being not essential. As expected, these barcodes can be used to quantify the abundance of different phage mutants in pools using BarSeq. However, in the current form these experiments seem added onto the manuscript without any real benefit. Different non-essential regions that could be targeted by barcode insertion have already been known before and a small barcode is hardly a difficult payload to insert, so this does not provide a significant advance and has otherwise nothing to do with the methodology that the authors present. In my view this section should either be removed or expanded in a way that connects it better to the rest of the work (e.g., by directly performing phenotypic selection experiments with pooled libraries of barcoded sgRNAs, if the authors like to use BarSeq for an approach similar to CRISPRi-Seq).*

We have addressed reviewer’s feedback by revising this section, detailing the goal of the experiment and improved general readability of the text.

The demonstration of inserting a 20-mer barcode with its primer binding region (required for Barseq) in the non-essential region of phages was never about the type of payload. The primary goal of this experiment is to demonstrate the importance of mapping of gene essentiality landscape at genome-wide scale and to show that this foundational work can lead to diverse sets of application, whether its inserting a payload or tracking engineered (barcoded) phages in a phage cocktails in different environmental contexts (such as in phage therapy applications or biocontrol measures in the agriculture, produce or meat production facilities). The importance of barcode use in diverse applications has been already discussed and reviewed in a number of publications including us (PMID: 29769716; 30659179; 35140226). As barcode/CRISPRi technologies have never been applied to phages as far as we know, we wanted to perform arrayed CRISPRi compared to pooled fitness assays to benchmark the technology so that future studies can be carefully designed.

We agree that pooled genome-wide CRISPRi can be employed, but as mentioned in the above comment, here we wanted to uncover any nuanced knockdown effects due to CRISPRi technology. We note here that, we have used CRISPRi and other barcoded technologies in the past (for example, PMIDs: 33048924; 34910616; 36805702; 29769716) in pooled fitness assay format and have noted enrichment of stronger fitness phenotype effects (“jackpot effects'') that might coverup some of the weaker/intermediate fitness traits. Especially when these technologies are applied to phages, we would have to be careful about how we interpret these jackpot effects due to lack of information on the genomic organization, operon architecture and transcription start sites in non-model phages. While this manuscript was under review, successful implementation of dCas13 based genome-wide pooled CRISPRi screen was reported for diverse phages by Adler et al (https://www.biorxiv.org/content/10.1101/2023.09.18.558157v1).

*The discussion section appropriately places the authors' work into context of the literature and the research problem as defined in the introduction section. Subsequently, the authors discuss different shortcomings of their approach and possible solutions or applications. These parts were interesting but - because they paraphrased a lot of content from the results section - could be easily streamlined and shortened.*

We believe it is important to discuss shortcomings of any technology, especially when the field is beginning to apply these new technologies to study phage biology. Detailing these shortcomings and to provide them in the context of prior knowledge in the results section was difficult. So even though some part of the discussion section may seem paraphrased from the results section, we think readers will refer to our work in better designing their CRISPRi assays and be vigilant about how to interpret and think about polarity effects while using CRISPRi of phages.

*The authors have included very nice supplementary notes with additional details that are of interest primarily for phage aficionados (greatly appreciated).*

Thank you!

*In my view this work definitely establishes genome-wide CRISPRi screens for bacteriophages, even though most of the technology has already been around and was used previously for phages (albeit certainly not at the same scale). Furthermore, even (kind-of) CRISPRi-Seq in a virus has already been reported previously for human cytomegalovirus (Hein and Weissmann, Nature Biotechnology 2021 (not cited)). I therefore feel that the authors need to provide additional arguments why their technology is a great advance compared to previous tools. This could be done in different ways. One possibility that comes to my mind would be pooled CRISPRi screens as suggested by the authors themselves in the discussion section to identify different conditionally essential genes in lambda and P1 (and possibly additional phages). Another possibility would be to show if / that the authors' technology can be applied to phages that are (unlike lambda or P1) highly resilient to DNA-targeting tools like*

*CRISPR-Cas. This is true for many phages including, e.g., well-studied lab phages T4 (due to covalent genome modification) and T5 (unknown mechanism) and could greatly limit the applicability of this technology.*

We thank the reviewer for kind appreciation of our work and critical feedback on the manuscript. We have cited Hein and Weissmann, Nature Biotechnology 2021 in this revised version.

As a technology, CRISPRi has been applied in numerous ways in the past on different host systems (human cell lines, bacteria, yeast etc) for studying viral infection pathways, antibiotic tolerance and mapping host gene essentiality in different genetic contexts. In each case, even though the same CRISPRi technology was used across different host systems, each system was unique and provided novel insights, thereby advancing the field of functional genomics.

In our opinion, all of these efforts are also crucial for the genetic screening field as they each help in vetting this new technology, benchmark the design criteria for different host/viral contexts, uncover the data analysis and interpretation challenges and also bring out something that we did not know earlier. This is specifically true when CRISPRI is applied to phages, because of their sheer diversity in gene content and genomic organization. Though CRISPRi has been used in other systems, it has never been applied to phage at the genome-wide scale (as appreciated by this reviewer).

Importantly, there has been only one study that used the CRISPR system to knock down one gene of phage lambda. This recent work used a naturally DNase-free CRISPR system (PMID 35659325; Cas12c system) for the knock down of a single phage gene, but it did not discuss or investigate the polarity issues we have uncovered here.

Finally, as mentioned above, while this manuscript was under review, successful implementation of dCas13 based genome-wide pooled CRISPRi screen (by some of us) was reported for diverse phages including T4 and T5 phages (Adler et al <https://www.biorxiv.org/content/10.1101/2023.09.18.558157v1>).

We sincerely believe that all CRISPR technologies are useful in different contexts or applications and future research will definitely address the advantages/benefits of one system over others in filling the knowledge gaps in phage functional genomics.

*Minor points:*

*1) The manuscript text in line 126 talks about the mcp gene of P1 in Figure 1 but the corresponding lane in the efficiency-of-plating assay is labeled 23. Even though this is explained in the figure legend I would suggest to directly label the EOP assay mcp instead of 23.*

Thanks. We have corrected the gene name.

*2) The authors show that a knockdown of ral has no phenotype because the indicator strain that they use has no active restriction-modification system (lines 172-174). It is for sure not critical but would be quite cool to show that this gene is conditionally essential for growth on a strain with an active restriction-modification system, highlighting the potential of the authors' technology for the investigation of phage-encoded anti-immunity factors.*

We thank the reviewer for this point.

We have performed *ral* essentiality experiment by assaying *ral* CRISPRi knockdown in two indicator *E. coli* strains that have an active type I restriction-modification system encoded by *hsdR-hsdM-hsdS* genes*.* Our results show that knockdown of *ral* is indeed conditionally essential.

We have included this following text in the main text.

Line:179

“The knockdown of *ral* (encoding a restriction inhibitor protein) does not result in a major defect in the EOP because our indicator strain lacks a functional type I restriction system[^55,56^](https://paperpile.com/c/BUmUl7/u5FyL+8N1WH). To probe the essentiality of *ral*, we repeated the knockdown assays on two different strains with active type I restriction system (Methods). These assays indicate the conditional essentiality of *ral* that depends on the genotype of the target bacterial strain (Supplementary Fig. 2).

***Reviewer #3:***

*This manuscript describes a method for performing CRISPRi on phage genes. A plasmid system for the expression of a catalytically inactive Cas12 and guide RNA molecules in E. coli was designed. Genes were targeted in two model E. coli phages, lambda and P1. The authors show that targeting of essential genes led to large r2012*

*eductions in phage replication while targeting non-essential genes generally did not affect replication. The difficulties in using this method on phages are highlighted.*

*This manuscript clearly demonstrates that the catalytically dead version of Cas12 can block phage replication. This is a result of interest, but not particularly surprising given previously published papers in this area. The problems with this approach for evaluating gene essentiality in phage genomes are important and predictable. Given the large number of polycistronic messages in phage genomes, polarity is bound to be a problem. This manuscript has value in confirming that polarity is a major problem for interpreting this type of experiment performed on phages.*

We would like to thank the reviewer for their kind words and comments on our work.

*While this manuscript contains data of interest, I do not think that it warrants publication in a journal at the level of PLoS Biology. To warrant publication, the authors would need to provide some mechanistic insight into how this CRISPRi system is functioning on the phage genome.*

This is the first genome-wide CRISPRi study on any phage (and here we have applied it to two widely used phages) and we believe our work is perfectly suited for publication in PLoS Biology. For instance, our work meets every word of this PLoS Biology “Methods and Resources Articles” requirement. ‘*It describes technical innovation, a novel approach to a previously inaccessible biological innovation/ substantial improvements over previously established methods. Resource articles consist of data sets or other significant scientific resources that are of general interest and provide exceptional value for the community that could spur future research.’*

This work is an important milestone in the phage functional genomics area and is of broad interest to the PLoS Biology readers and to the larger research community. There has been an increasing interest in understanding how phages impact microbiome dynamics, virulence and evolution. However, there are no methods for doing genome-wide phage functional genomics that might start providing much needed insights on the role of different genetic content. Towards the larger goal of understanding phage genomic architecture, new technologies based on CRISPR are being developed to do phage engineering (PMID 36316451). As thousands of new phages are isolated and sequenced each day, using classical methods such as creating amber mutations will never scale to thousands of phages for performing genome-wide mapping of essential phage gene functions.

We strongly believe that our work will inspire a future generation of phage biologists to apply CRISPRi technology across diverse phages and start to fill the current technology and knowledge gaps in the field of phage functional genomics. Our systematic study of polarity effects in phage CRISPRi assays will also provide important insights and cautionary tale to phage practitioners as they apply these new technologies to new or non-model phages.

We believe that the methods presented here will enable phage biologists to use CRISPRi technology in their daily phage functional genomics tasks. A simple question of whether a particular phage gene is needed for phage infection and if that can be deleted without losing phage infectivity can be addressed with a simple crRNA cloning and testing it in the lab.

We are excited about the potential of the CRISPRi approach in phage biology research.

*While they hypothesize about the effects of polarity, no experiments are performed to address this issue (e.g. RT-qPCR). As presented, I would conclude that CRISPRi is really not a good method to assess essentiality in phages. This manuscript makes the problems evident, but does not provide insight as to how these problems might be overcome.*

The main goal of our work was to apply CRISPRi to two model phages at genome-wide scale in an arrayed format so that we avoid any jackpot effects of stronger fit phenotypes and demonstrate the feasibility of the method. Another goal was to provide a resource for phage engineers to develop ways to engineer phages with different payloads in non-essential genetic content without compromising phage fitness. Along the way, we uncovered the polarity effects in our dataset, and polarity as an effect has been reported for every genetic tool applied in bacterial systems so far (Including some widely used resources, for example: https://journals.asm.org/doi/10.1128/msystems.00813-21).

The polarity of Nin genes in lambda has been demonstrated unambiguously in prior studies using classical genetics and how antiterminators overcome the polarity issue (for example: PMID 7672588, 6219042, 5344297, 25742714). Here we uncover the polarity of Nin genes in CRISPRi assays and overcome it by functional complementation of *Q* gene in trans while targeting Nin region. We reiterate that our CRISPRi knockdown assays are highly consistent with literature in cataloging other non-essential genes in lambda and P1 phages.

As we have presented the importance of our work in the larger context of the phage functional genomics field (in the Introduction and Discussion sections), we would like to point out that, there are currently no methods that (are easy, and economical) can be extended to genome-wide scale for mapping phage gene essentiality. Considering the effect of polarity as a commonly observed phenomenon, we believe that the CRISPRi method is more reliable in classifying genes as non-essential than as essential. We have explicitly presented this point as below:

Line:276

“Finally, this study uncovers the polar effect of CRISPRi in phages. We recommend using CRISPRi for mapping non-essential regions while caution towards interpreting essential gene assignments when applied to less studied phages where transcripts have not yet been mapped.”

While this manuscript was under review, two papers using dCas13 based genome-wide pooled CRISPRi screens came out. One was (by some of us) reported for dCas13 CRISPRI for diverse phages including T4 and T5 phages (Adler et al <https://www.biorxiv.org/content/10.1101/2023.09.18.558157v1>); while other one used dCas13 to study regulation within synthetic operons (https://www.biorxiv.org/content/10.1101/2023.10.11.561958v1). These authors report considerable reduced polarity effects when dCas13 used compared to dCas9. In this context, we believe the future studies comparing these different CRISPRi systems on polarity effects and efficiency of knockdowns with different phage encoded antiterminators provide much needed insights.
